# Isolation and Identification of Two Novel Attractant Compounds from Chinese Cockroach (*Eupolyphaga sinensis* Walker) by Combination of HSCCC, NMR and CD Techniques

**DOI:** 10.3390/molecules180911299

**Published:** 2013-09-13

**Authors:** Wei Jiang, Xiaodan Wu, Bin Wu

**Affiliations:** 1 Ocean College, Zhejiang University, Hangzhou 310058, China; E-Mail: jw6912@163.com; 2 Center of Analysis and Measurement, Zhejiang University, Hangzhou 310058, China; E-Mail: wxd_zju@163.com

**Keywords:** attractants, counter-current chromatograph, ground beetles, *Eupolyphaga sinensis* Walker

## Abstract

High-speed counter-current chromatography (HSCCC) with a two-phase solvent system composed of *n*-hextane-ethyl acetate-methanol-water (1.5:1:1.5:1, *v/v/v/v*) was applied to the isolation and purification of attractants from Chinese cockroach, *Eupolyphaga sinensis* Walker. Two new attractants with attractant activity towards the male insects were obtained from the extract sample in a one-step separation. Their purities were determined by HPLC. Subsequent MS, NMR and CD analyses have led to the characterization of (*R*)-3-ethyl-6,8-dihydroxy-7-methyl-3,4-dihydroisochromen-1-one (**1**) and (*R*)-6,8-dihydroxy-3,7-dimethyl-3,4-dihydroisochromen-1-one (**2**), two novel isocumarin type attractants. Based on these results, it is concluded that HSCCC is a viable separation method option for purifying insect attractants, while effectively maintaining the attracting activity of the isolates. This is the first attempt to apply counter-current chromatography technique to separate attractants from Chinese cockroach.

## 1. Introduction

**Figure 1:**
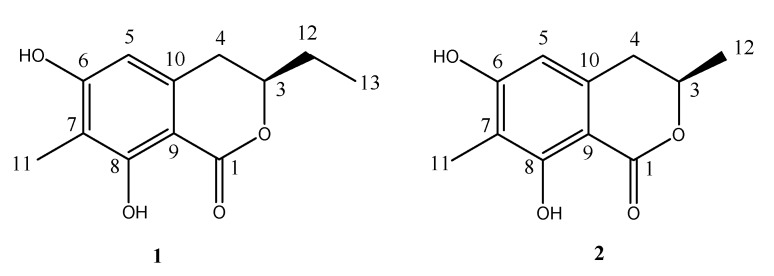
Structures of new attractants from *Eupolyphaga sinensis* Walker.

Chinese cockroach or ground beetles, *Eupolyphaga sinensis* Walker (Blattaria: Polyphagidae), an insect species which is used as material in Traditional Chinese Medicines are found in many parts of China [1]. *E. sinensis* occurs in a wide variety of climatic conditions, from subtropical areas in the south to temperate climates in the northwest [2]. Like many other insects, cockroaches utilize volatile attractants produced by females to attract males [3]. Some of the attractants were proved to act as pheromones [4], that accumulate on the surface of the female’s cuticle [5]. Some sex pheromones of cockroaches have been reported, such as periplanone D from smokybrown cockroaches, and periplanone A and B from American cockroaches [6,7,8,9]. The sex pheromone of the German cockroach, *Blattella germanica*, has been characterized as gentisyl quinone isovalerate, which provides a new tool in cockroach population detection, monitoring, and control [10]. However, attractants of the Chinese cockroach, *Eupolyphaga sinensis* Walker (Blattaria: Polyphagidae) have never been purified and identified. The traditional methods of isolation and purification attractants are preparative gas chromatography [10,11], gel-filtration chromatography, reversed-phase high-performance liquid chromatography [12], ion-exchange chromatography [13], solid-phase microextraction [14]. Although a variety of chromatographic systems have been used to separate and analyze attractants, isolation and purification of insect attractants are still a tough task for researchers owing to the difficulty of maintaining the attracting activity. High-speed counter-current chromatography (HSCCC) is an efficient preparative technique widely used for the separation and purification of natural and synthetic products [15,16]. Counter-current chromatography benefits from great advantages when compared with the traditional liquid–solid separation methods in separation and purification of insect sex pheromones: eliminating the complications resulting from the solid support matrix, such as irreversible adsorptive sample loss and deactivation, tailing of solute peaks, and contamination. The instability of the pheromones is always an obstacle for their separation [10]. This technique has an advantage over HPLC in the separation of sex pheromones since there is no packing material nor metal plumbing involved which can cause metal oxidation and sample adsorption or degradation. Although preparative gas chromatography also could exclude metal oxidation and degradation of pheromones, it is difficult to obtain enough samples for NMR and CD analysis. In order to obtain sufficient amounts of compound to elucidate the absolute structures and to exclude the possibility of losing male attracting activities after the process of separation, in the present work, a HSCCC protocol was established for simultaneous isolation and purification of two attractants from *Eupolyphaga sinensis* Walker which are candidate pheromones. The attractants’ planar structures were identified by 1D and 2D NMR and MS means and absolute structures were identified by circular dichroism (CD) analysis. Two novel attractants were identified as (*R*)-3-ethyl-6,8-dihydroxy-7-methyl-3,4-dihydroisochromen-1-one (**1**) and (*R*)-6,8-dihydroxy-3,7-dimethyl-3,4-dihydroisochromen-1-one (**2**), as illustrated in Figure 1. In this paper, we present a first attempt to use counter-current chromatography technique to separate attractants and to maintain attracting activity during separation process.

## 2. Results and Discussion

### 2.1. HSCCC of the New Attractants

Selection of the two-phase solvent system was the key point in the HSCCC separation. In order to achieve an efficient resolution of the target attractants, *n*-hexane-ethyl acetate-methanol-water and petroleum ether-ethyl acetate-methanol-water were used as the two-phase solvent systems for the preliminary studies. Seven different HSCCC solvent systems were examined. According to the previous literature, an ideal two-phase solvent system should meet the following requirements: (i) relatively short settling time; (ii) sufficient sample solubility; (iii) no sample decomposition; (iv) suitable partition coefficient *K* values of the target compound (usually between 0.5 and 2) (Table 1); (v) satisfactory separation factor (α) between two components (>1.5). α = *K*B/*K*A, *K*B > *K*A; (vi) good retention time of the stationary phase [17,18]. *K* values calculated for three *n*-hexane-ethyl acetate-methanol-water and four petroleum ether-ethyl acetate-methanol-water systems, expressed as the peak area of target component in the upper phase divided by that in the lower phase are shown in Table 1. 

**Table 1:** The *K*-values and separation factors of the target compounds in several solvent systems.

Solvent system(*v/v/v/v*)	*K*_UP/LP_ *^a^*		α
	*K*A *^b^*	*K*B *^c^*	
*n*-hexane-ethyl acetate-methanol-water(1:1:1:1)	1.61	3.13	1.94
*n*-hexane-ethyl acetate-methanol-water(1.5:1:1.5:1)	0.68	1.26	1.85
*n*-hexane-ethyl acetate-methanol-water(2:1:2:1)	0.33	0.57	1.73
petroleum ether-ethylacetate-methanol-water(1:1:1:1)	0.89	1.75	1.97
petroleum ether-ethylacetate-methanol-water(1.8:1:1.8:1)	0.17	0.40	2.35
petroleum ether-ethylacetate-methanol-water(1.5:1:1.5:1)	0.25	0.55	2.20
petroleum ether-ethylacetate-methanol-water(2:1:2:1)	0.13	0.29	2.23

*^a^* Expressed as the peak area of target components in the upper phase divided by that in the lower phase. *^b^* *K* value of compound **2**. *^c^ K* value of compound **1**.

**Figure 2:**
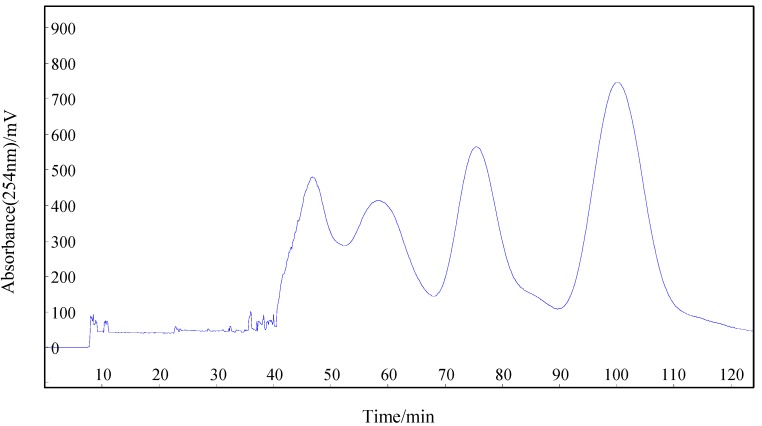
HSCCC separartion of the crude extract (**1**) attractant **1**; (**2**) attractant **2**. HSCCC separation conditions: column rotary speed, 800 rpm; column temperature, 25 °C; solvent system, *n*-hexane-ethyl acetate-methanol-water (1.5:1:1.5:1, *v/v/v/v*); mobile phase, lower phase; flow rate, 2 mL/min; detection 254 nm; retention of the stationary phase, 80%; sample injected: 160 mg in 3 mL upper organic phase and 3 mL lower aqueous phase; separation mode: head-to-tail.

Suitable *K* values and sufficient peak resolution of HSCCC was achieved at the volume ratio of 1.5:1:1.5:1 (see Table 1). When this selected two-phase solvent system was used for the separation, separation of the target compounds was achieved with satisfactory peak resolution and retention times (101 min for compound **1** and 77 min for compound **2**). The HSCCC chromatogram is shown in Figure 2. Crude extract (160 mg) was injected and the HSCCC separation was performed in head-to-tail elution mode. The mixture eluted after the target compounds were removed by pumping out the stationary phase so that solvents and time could be saved. Each HSCCC peak fraction was analyzed by HPLC and the chromatograms are shown in Figure 2B and Figure 2C. As a result, 7.1 mg of compound **1** and 5.3 mg of compound **2** with the purity >95% (determined by HPLC) for both isolates, were obtained in a one-step separation.

### 2.2. HPLC Analyses of Fractions

**Figure 3:**
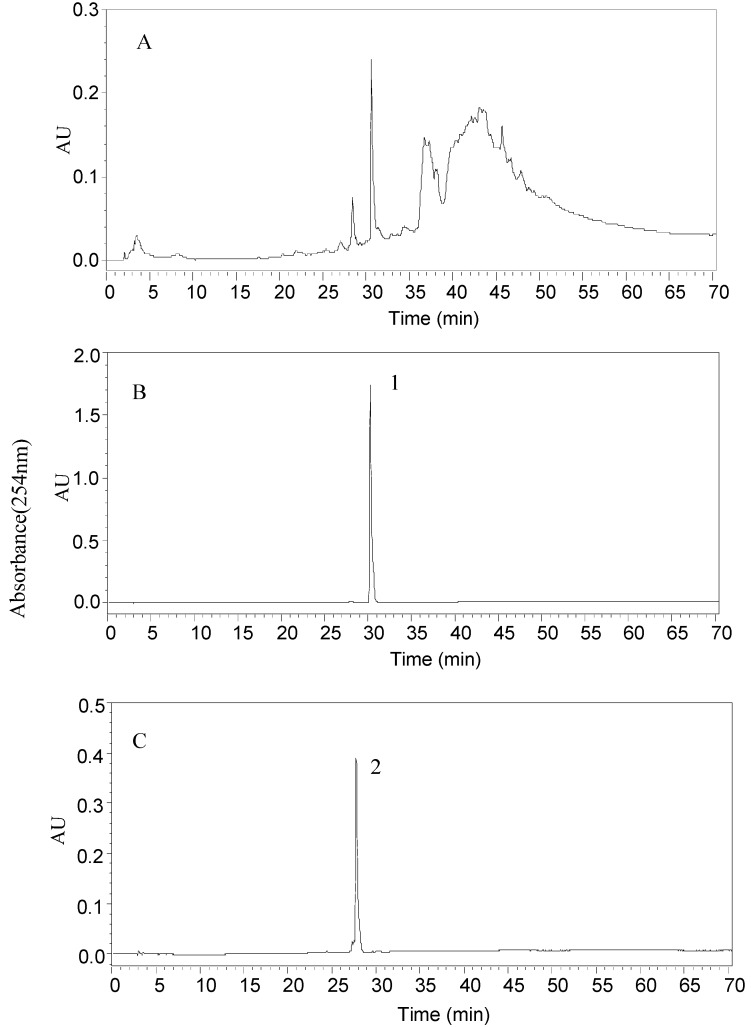
HPLC chromatograms (**A**) CHCl_3_ fraction; (**B**) HSCCC fraction of peak 1 (**C**); HSCCC fraction of peak 2. Conditions: column, Zorbax SB-C18 column (250 mm × 4.6 mm, I.D., 5 µm, Agilent); column temperature, 25 °C; mobile phase, methanol and water at the gradient: methanol, 0–30 min, 20%–100%, 30–70 min, 100%; flow rate, 0.8 mL/min; detection, 254 nm.

To define the optimal HPLC condition for separation the attractants, the effects of eluted mode, mobil phase, flow rate, detection wavelength and column temperature were investigated. An Agilent Zorbax SB-C18 column (250 × 4.6 mm, I.D., 5 μm) was used. Methanol (HPLC-grade) was obtained from Tedia (Fairfield, OH, USA). Water was purified with a Mili-Q academic water purification system (Millipore, Bedford, MA, USA). Two target compounds (30 min for compound **1** and 27 min for compound 2) were separated using the following eluted mode: 0~30 min, 20%~100% methanol, 30–70 min, 100% methanol. The flow rate was 0.8 mL/min and the column temperature was 25 °C. The effluent was monitored at 254 nm. The HPLC chromatogram of the crude CHCl_3_ extract is presented in Figure 3A and shows that two main peaks were mixed with baseline noise and several peaks. The target compounds from two HSCCC fractions were dried under vacuum, redissolved in methanol and subjected to analysis under the same HPLC conditions as that for the crude sample, whose chromatograms were shown in Figure 3B and C. The results showed that peak 1 and peak 2 were identical to attractant **1** and attractant **2** and had satisfactory purity.

### 2.3. Structural Elucidation of the New Attractants

**Figure 4:**
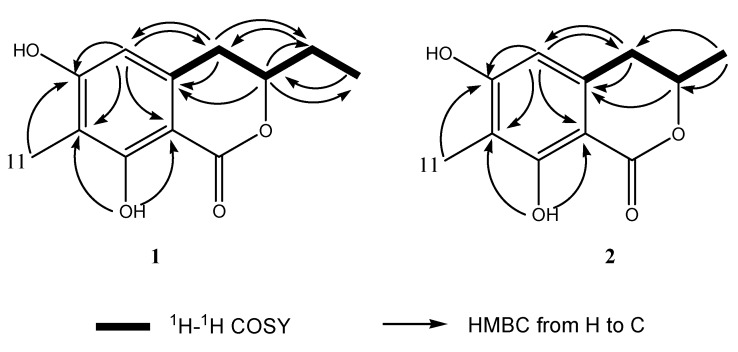
Key ^1^H ^1^H COSY and HMBC correlations of compounds **1** and **2**.

Compound **1** was obtained as a white powder. The HR-TOF-MS (positive CI full-scan mode, CH_4_ as reagent gas) exhibited a molecular ion peak at *m/z* 222.0898 (calcd. 222.0892), corresponding to the molecular formula C_12_H_14_O_4_ (see Supporting information S14). The UV spectrum showed typical benzenoid absorptions at 220 and 270 nm. The phenolic nature of the compound was also indicated by its characteristic colour reactions (FeCl_3_: purple; phosphomolybdic acid reagent: deep blue). The ^13^C-NMR spectrum (Table 2) displayed 12 signals, among which six were aromatic, and the remaining seven were aliphatic carbon signals with a ester carbonyl resonance, indicating a coumarin or isocoumarin as further confirmed by the molecular formula C_12_H_14_O_4_. The 3,4-dihydroisocoumarin skeleton of **1** was deduced from analysis of resonances in the ^13^C-NMR spectrum (Table 2) at *δ*_C_ 170.8 (*s*), 32.4 (*d*) and 80.7 (*t*) attributable to C-1, C-3 and C-4, and confirmed by the ^1^H-^1^H COSY correlation from H-4 to H-3 (Figure 4), and the HMBC correlations from H-3 and H-4 to the aromatic C-10. ^1^H ^1^H COSY correlation from H-12 to Me-13 and H-3 and HMBC correlation from Me-13 to C-3 proved the presence of ethyl pendant unit at C-3. The methyl group was deduced to attach at C-7 by analysis the HMBC correlations from the protons of Me-11 to oxygenated aromatic carbon atom C-6. A sharp singlet at *δ_H_* 11.40 attributable to a hydrogen bonded phenolic OH was observed in the ^1^H-NMR spectrum of **1**, revealing that one of the hydroxyl groups was attached at C-8. The remaining OH group was deduced to be at C-6 from the HMBC correlation between aromatic H-5 and hydroxylated C-6. From the above information, compound **1** was deduced to be a new 3,4-dihydroisocoumarin, 3-ethyl-6,8-dihydroxy-7-methyl-3,4-dihydroisochromen-1-one. Although the planar structure can be elucidated by analysis of 1D and 2D NMR and MS data (see Supporting information, Figures S1–S7 and S15), the absolute configuration of **1** could not be determined from the above information.

**Table 2:** NMR Data (500 MHz) for compounds **1** and **2** in CDCl_3__._

Position	1			2	
	δ_C_ *^a^*^,*b*^	δ_H_ *^c^* mult. (*J* in Hz)		δ_C_ *^a^*^,*b*^	δ_H_ *^c^* mult. (*J* in Hz)
1	170.8 (s)			170.4 (s)	
3	80.7 (d)	4.43 (dddd, *J* = 10.5, 6.5, 5.0, 4.0)		75.7 (d)	4.63 (m)
4	32.4 (t)	2.80 (m)		34.5 (t)	2.83 (m)
5	106.0 (d)	6.26 (s)		105.7 (d)	6.22 (s)
6	160.7 (s)			160.3 (s)	
7	109.9 (s)			109.8 (s)	
8	162.1 (s)	11.40 (OH)		162.2 (s)	11.46 (OH)
9	101.2 (s)			101.3 (s)	
10	138.1 (s)			138.0 (s)	
11	7.5 (q)	2.12 (s)		7.4 (q)	2.13 (s)
12	27.8 (t)	1.77 (m), 1.87 (m)		20.7 (q)	1.51 (d, *J =* 6.5)
13	9.3 (q)	1.06 (t, *J =* 7.5)			

*^a^* Recorded on 125 MHz. *^b^* Multiplicities inferred from DEPT and HMQC experiments. *^c^* Recorded on 500 MHz.

Compound **2** was isolated as a white powder. The molecular formula was determined to be C_11_H_12_O_4_ by analysis of the HR-TOF-MS (positive CI full-scan mode, CH_4_ as reagent gas) ion peak at *m/z* 208.0744 (calcd. 208.0736) (see Supporting information S15). Compound **2** also exhibited characteristic phenolic colour reactions. The ^1^H- and ^13^C-NMR spectra of **2** showed similar chemical shifts and the same multiplicities for most carbon atoms as in **1** with minor differences, indicating a 3,4-dihydroisocoumarin skeleton with two methyl, two hydroxy group in the structure of **2**. HMBC correlations completed the definition of all the functional groups in compound **2** (Figure 4). The long-range correlations from the Me proton at *δ*_H_ = 1.51 (d*, J =* 6.5 Hz, Me-12) to the carbon signals at *δ*_C_ = 75.7 (d, C-3) and 34.5 (t, C-4) revealed a methyl group attaching at C-3. The planar structure of compounds **2** was elucidated as the same as the known isocoumarin, monaschromone, isolated from the fungus *Monascus purpureus* [19].

**Figure 5:**
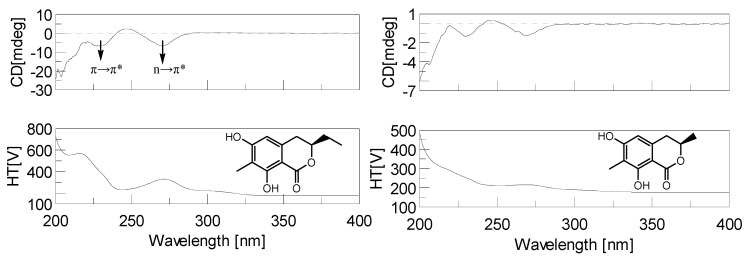
The CD spectra of attractants 1 and 2.

The chirality of insect attractants is crucial to their attracting activity [20]. In order to elucidate the absolute structure of compound **1** and **2**, the isolates from HSCCC separation was subjected to CD experiments. The CD spectra of **1** and **2** are showed in Figure 5. Chiroptical properties of dihydroisocoumarins’ benzoic ester chromophores have been systematically investigated [21]. It is believed that the Cotton effect of n→n* origin could be used for establishing the absolute conformation of the hetero ring of this chromophore system, since the sign of this type of Cotton effect is independent of the substitution pattern of the aromatic ring system [22]. The CD measurements of two attractants (**1** and **2**) were carried out in the UV absorption region as shown in Figure 5.

**Figure 6:**
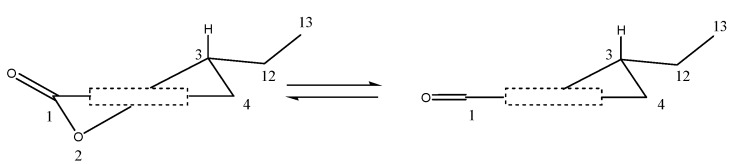
Standard projection from the aromatic into the heterocyclic ring of **1** (half-chair and boat conformation).

In the CD spectra of compounds **1** and **2**, three Cotton effects were observed. The bands of negative sign around 270 nm could be unequivocally assigned to the n→π* transition of the carbonyl group on the basis of the characteristic hypsochrome shifts. The heterocyclic rings of **1** and **2** were deduced to be in a half-chair or a chair conformation, in which the ethyl or methyl group at C-3 is oriented equatorially as illustrated in Figure 6, based on the helicity rule of the chiral benzoic ester chromophore of the n→n* band of conformationally fixed dihydrocoumarin derivatives [19]. From above CD analysis and the comparison with literature values [23], the absolute configurations at C-3 of compounds **1** and **2** could be deduced as *R*.

These inferences were also confirmed by their molecular rotations (−87.4 for compound 1 and −76.2 for compound **2**), when compared with those of (*R*)-(−) mellein, (*S*)-(+) mellein [24] and nonaschromone ((3*S*)-3,4-dihydro-6,8-dihydroxy-3,7-dimethyl-1*H*-isochromen-1-one) in the literature [17]. Although the planar structure of compounds **2** is the same as that of the known compound monaschromone which exhibited a positive Cotton effect at 270 nm in its circular dichroism spectrum, revealing a 3*S* configuration [13], attractant **2** was proved to be a new compound based on the absolute configuration analysis. After analyzing all the relevant information, it could be concluded that two HSCCC isolates were two new isocoumarins, (*R*)-3-ethyl-6,8-dihydroxy-7-methyl-3,4-dihydroisochromen-1-one (**1**) and (*R*)-6,8-dihydroxy-3,7-dimethyl-3,4-dihydroisochromen-1-one (**2**).

### 2.4. Attracting Activity

Attracting activity bioassay was conducted in a disposable Petri dish modified from Guss *et al.* [25]. The result showed that compounds **1** and **2** on a filter paper can attract male ground beetles in 5 min, which indicated that attractants from the HSCCC separation process maintained their bioactivity.

## 3. Experimental

### 3.1. Apparatus

High-speed counter-current chromatography (HSCCC) was performed using a Model TBE-300B high-speed counter-current chromatograph (Tauto Biotech Co. Ltd., Shanghai, China). The apparatus with the maximum rotational speed of 1,000 rpm was equipped with three polytetrafluoroethylene preparative coils (ID 2.6 mm, total volume is 300 mL) and a 20 mL manual sample loop. The β values of the multilayer coil varied from 0.5 at internal terminal to 0.8 at the external terminal. An integrated AKTA prime system (GE, Fairfield, CT, USA) was used to pump the two-phase solvent system, measure the UV absorbance of the effluent at 254 nm, and collect the fractions. An HX 105 constant temperature regulator (Beijing Boyikang Lab Instrument, Beijing, China) was used to control the separation temperature. And a N2000 data analysis system (Institute of Automation Engineering, Zhejiang University, Hangzhou, China) was employed for data collection and analysis. The high-performance liquid chromatography (HPLC) system used was composed of a Waters 717 plus Autosampler, a Waters 600 Controller, a Waters 996 Photodiode Array Detecter and a Waters Millog workstation. Optical rotations were recorded on a Perkin-Elmer-341 polarimeter. ^1^H-NMR (500 MHz) and ^13^C-NMR (125 MHz) spectra were measured at 25 °C on a Bruker AVANCE DMX 500 NMR spectrometer with TMS as internal standard. TOF-MS were recorded on a GCT-Premier GC-TOF-MS spectrometer. ESIMS were recorded on an Agilent 6460 Triple Quad LC/MS. ECD spectra were recorded on JASCO J-815 circular dichroism spectrometer.

### 3.2. Reagents

The organic solvents used in HSCCC separation were of analytical grade purchased from Sayfo Technology (Tianjin, China) and chromatographic grade in HPLC analysis purchased from Tedia (Fairfield, OH, USA). Deionized water used for all solutions and dilutions was prepared by reverse osmosis using a Milli-Q (18 MΩ) system (Millipore, Bedford, MA, USA).

### 3.3. Insect Material

Cuticle from pubertal female ground beetles was used as a raw material source of the attractants. Samples of *Eupolyphaga sinensis* Walker were collected in Changzhou County, Jiangsu Province, China, and identified by Prof. Changxi Zhang (Jinhua Medical College, Jinhua, China). A voucher specimen (Vw281) is maintained at the Jinhua Medical College.

### 3.4. Preparation of Crude Sample

The dried cuticles (5 kg) of pubertal female *E. sinensis* were extracted at room temperature three times with methanol (3 × 5 L). The extracts were evaporated *in vacuo* to afford a gummy residue (12.1 g). This residue was partitioned in H_2_O (1 L) and extracted with petroleum ether (4 × 1 L), CHCl_3_ (4 × 1 L) and EtOAc (4 × 1 L), successively. Three main fractions were evaporated *in vacuo* to afford a gummy petroleum ether fraction residue (2.2 g), a gummy CHCl_3_ fraction residue (1.0 g) and a gummy EtOAc fraction residue (1.4 g). Small samples of the fractions were submitted to testing of male attracting activity, with the gum obtained from the CHCl_3_ fraction showing bioactivities.

### 3.5. Preparation of the Two-phase Solvent Systems and Sample Solution

*n*-Hexane-ethyl acetate-methanol-water and petroleum ether-ethyl acetate-methanol-water were used as the two-phase solvent systems for optimization. In order to find a suitable volume ratio, the partition coefficient was determined by HPLC analysis. Seven mixtures of solutions were made according to the method described by He *et al.* [15]. About 1 mg of crude sample was added to a test tube containing 2 mL of each phase of each of the two-phase solvent systems. The tubes were shaken vigorously for 1min and solutions were thoroughly mixed. The mixture was equilibrated after 10 min and divided into two phases. Then equal volumes of upper and lower phases were evaporated *in vacuo* to dryness separately. The residues dissolving in methanol were subject to HPLC analysis. Measurement of the peak areas afforded calculated partition coefficient (*K*) values of the target compounds. The *K* value was showed as a peak area of the compounds in the stationary phase divided by the peak area of the compounds in the mobile phase (Table 1).

### 3.6. HSCCC Separation Procedure

The optimized mixtures for the HSCCC purification were prepared in a separation funnel and thoroughly equilibrated at room temperature, whose upper phase was used as a stationary phase and the lower phase as a mobile phase. The crude sample was dissolved in the selected two-phase solvent system. The HSCCC preparation was carried out in the following steps: First, the coil columns were filled with upper solution as the stationary phase. Then, the sample solution was injected into the sample loop and the lower solution was pumped as the mobile phase in the mode of head-to-tail elution. The flow rate, column rotary speed and effluent detection wavelength were set at 2 mL/min, 800 rpm and 254 nm. The target compounds were obtained according to the eluted profile only in a one-step separation.

### 3.7. HPLC Analysis and Identification of HSCCC Peak Fractions

The crude CHCl_3_ extract and each fraction obtained after HSCCC separation were analyzed by HPLC. HPLC analysis of the crude sample and HSCCC peak fractions were performed with a Zorbax SB-C18 column (250 mm × 4. 6 mm, I.D., 5 µm, Agilent). The mobile phase was composed of methanol and water at the gradient: methanol, 0–30 min, 20%–100%; 40 min, 100%. The flow rate was 0.8 mL/min and the effluent was monitored on-line at 254 nm. Identification of each HSCCC peak fraction was performed by combination of spectroscopic means. MS analyses were performed by high-performance liquid chromatography (HPLC) coupled with diode-array (DAD) and electrospray ionization (Agilent 6460 Triple Quad LC/MS). NMR analyses were performed on a Bruker 500 MHz spectrometer. HR-TOF-MS analyses were performed on GCT-Premier GC-TOF-MS spectrometer. Circular dichroism analyses were performed on JASCO J-815 circular dichroism spectrometer.

### 3.8. Attracting Activity Assay

Attracting activity bioassay was conducted according to Guss’s method [25]. Five male ground beetles were placed in a disposable Petri dish (10 × 1.5 cm) and acclimated for 30 min. Then test compounds or fractions in 10 μL (approximately 3 μg of each compound from ten insects) of hexane were applied to a filter paper chip (5 mm^2^). After solvent evaporation, the treated chip was placed in the Petri dish. Positive responses of orientation of the male insects toward the chip were observed and recorded.

## 4. Conclusions

The instability of pheromones makes their separation and characterization difficult [10]. Chromatographic separation of insect pheromones is difficult by conventional methods. Although counter-current chromatography has been successfully applied to the separation of various natural product components, this is the first time that it has been introduced for purification of insect attractants. In the present study, two novel attractants from Chinese cockroach were successfully purified using the HSCCC technique and their structures, including absolute stereochemistry, were determined using NMR, MS and CD spectroscopic methods. The advantages over traditional separation methods for attractants can be summarized as follows: (i) Since it only required one day to purify two compounds after extraction, HSCCC can significantly shorten separation time and steps; (ii) because of the support-free partition system, decomposition of attractants which are vulnerable to solid separation materials can be avoided; (iii) since the support-free partition system has no adsorptive loss of the target compounds, it is possible to isolate sufficient amounts of minor attractants for structural elucidation without adsorptive losses. From the results of the present study, HSCCC technique is proved to be an option for purifying attractants.

## Supplementary Materials

Supplementary materials can be accessed at: http://www.mdpi.com/1420-3049/18/9/11299/s1.
