# Supplementary material for: Isolation and Identification of Two Novel Attractant Compounds from Chinese Cockroach (Eupolyphaga sinensis Walker) by Combination of HSCCC, NMR and CD Techniques
Source: Molecules. 2013 Sep 13;18(9):11299–310. doi: 10.3390/molecules180911299 (PMC6270134; doi:10.3390/molecules180911299)

Supplementary Materials

Contents

Figure S1. ^{1}H-NMR in $CDCl_3$ for compound **1**.
Figure S2. ^{13}C-NMR in $CDCl_3$ for compound **1**.
Figure S3. DEPT in $CDCl_3$ for compound **1**.
Figure S4. COSY in $CDCl_3$ for compound **1**.
Figure S5. HMQC in $CDCl_3$ for compound **1**.
Figure S6. HMBC-1 in $CDCl_3$ for compound **1**.
Figure S7. HMBC-2 in $CDCl_3$ for compound **1**.
Figure S8. ^{1}H-NMR in $CDCl_3$ for compound **2**.
Figure S9. ^{13}C-NMR in $CDCl_3$ for compound **2**.
Figure S10. DEPT in $CDCl_3$ for compound **2**.
Figure S11. COSY in $CDCl_3$ for compound **2**.
Figure S12. HMQC in $CDCl_3$ for compound **2**.
Figure S13. HMBC in $CDCl_3$ for compound **2**.
Figure S14. HR-TOF-MS for compound **1**.
Figure S15. HR-TOF-MS for compound **2**.
Figure S16. CCC chromatogram.
Figure S17. CD spectrum for compound **1**.
Figure S18. CD spectrum for compound **2**.

Figure S1. ^{1}H-NMR in $CDCl_3$ for compound **1**.

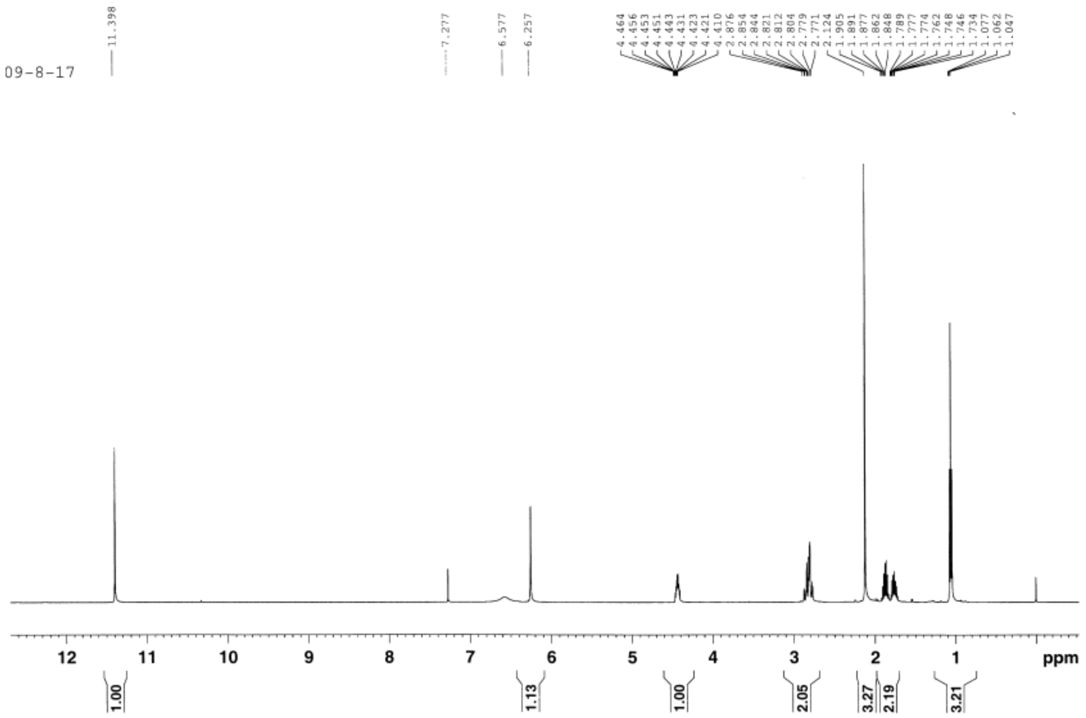

Figure S2. ^{13}C-NMR in $CDCl_3$ for compound **1**.

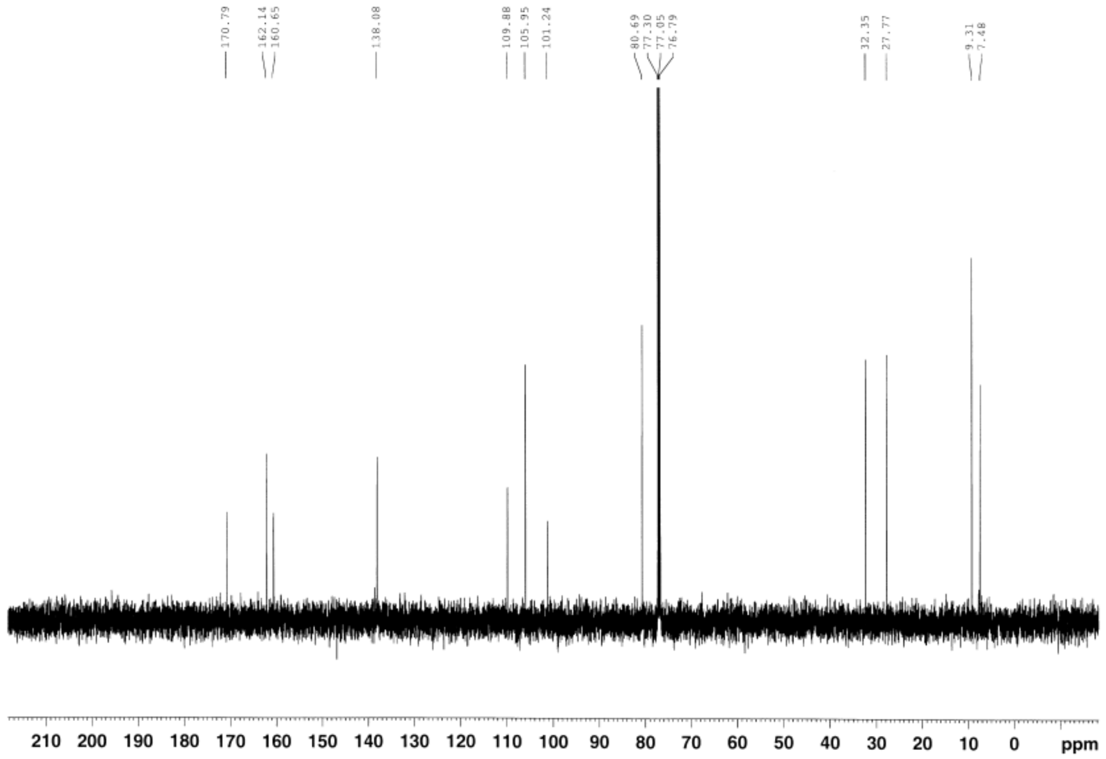

Figure S3. DEPT in $CDCl_3$ for compound **1**.

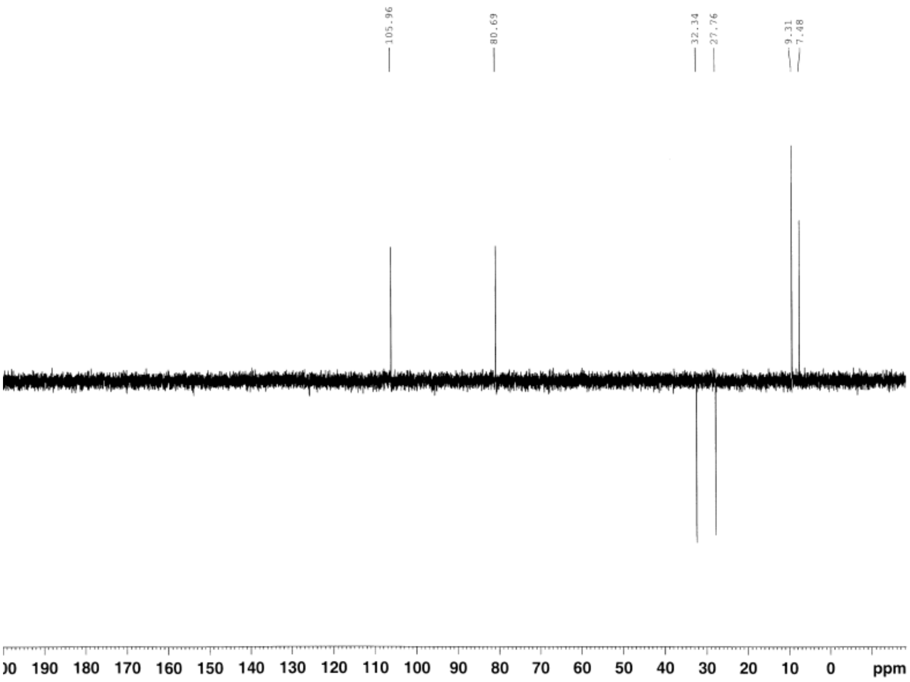

Figure S4. COSY in $CDCl_3$ for compound **1**.

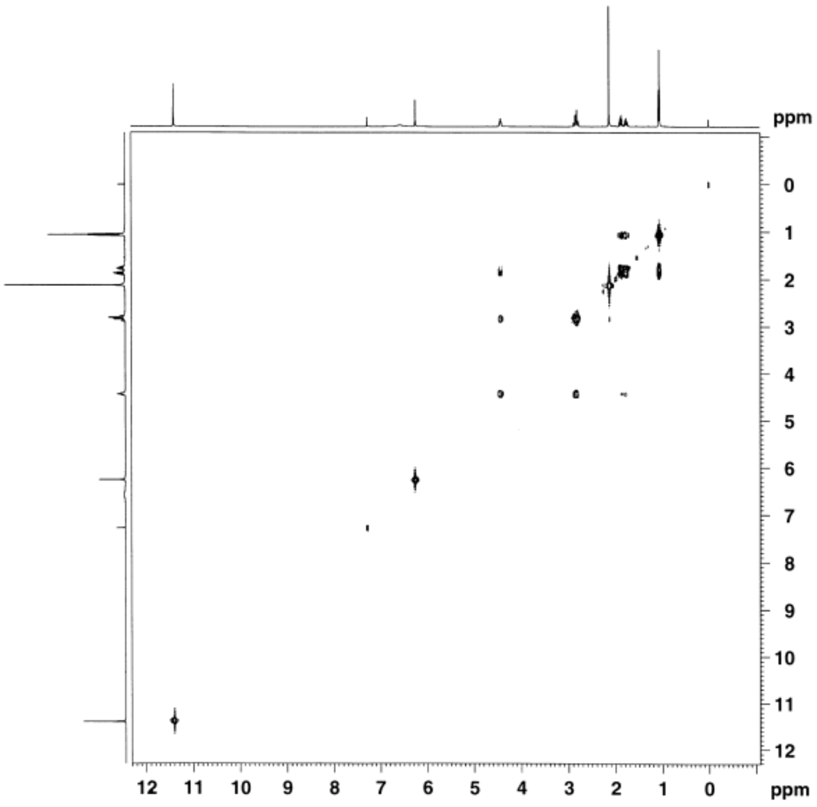

Figure S5. HMQC in $CDCl_3$ for compound **1**.

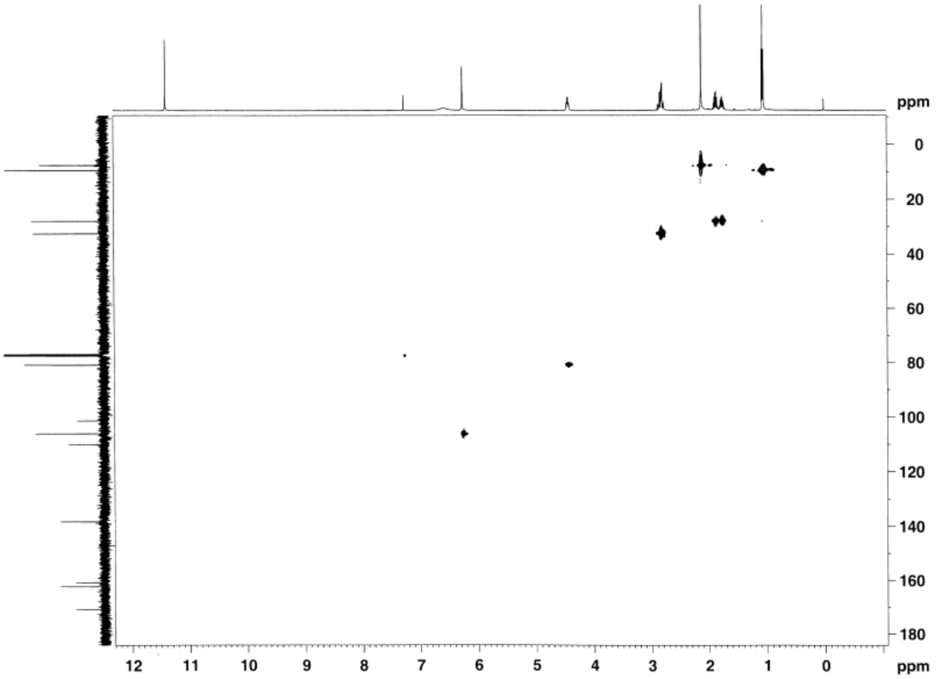

Figure S6. HMBC-1 in $CDCl_3$ for compound **1**.

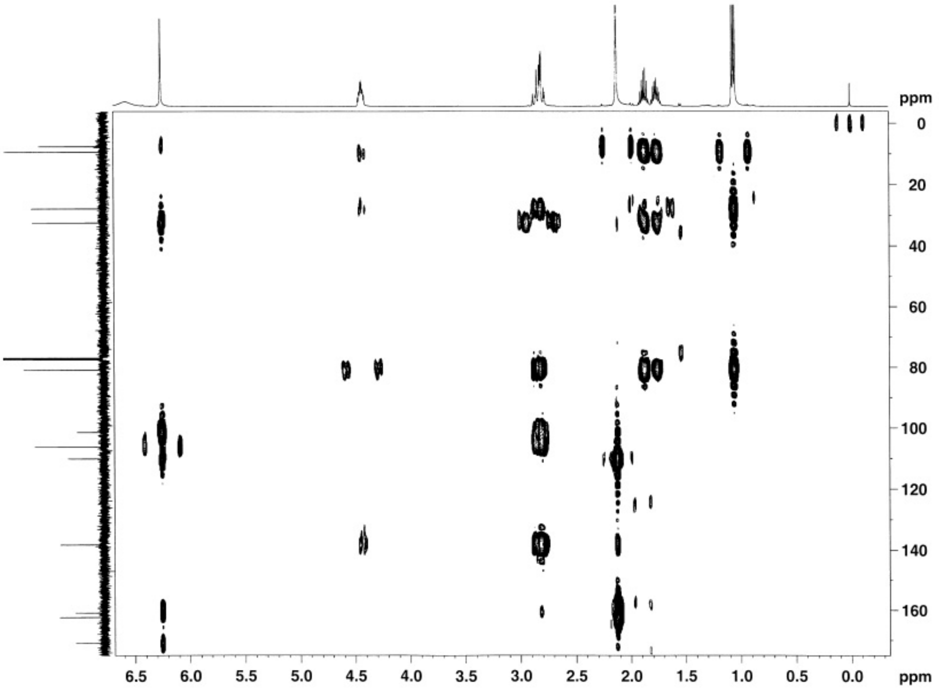

Figure S7. HMBC-2 in $CDCl_3$ for compound **1**.

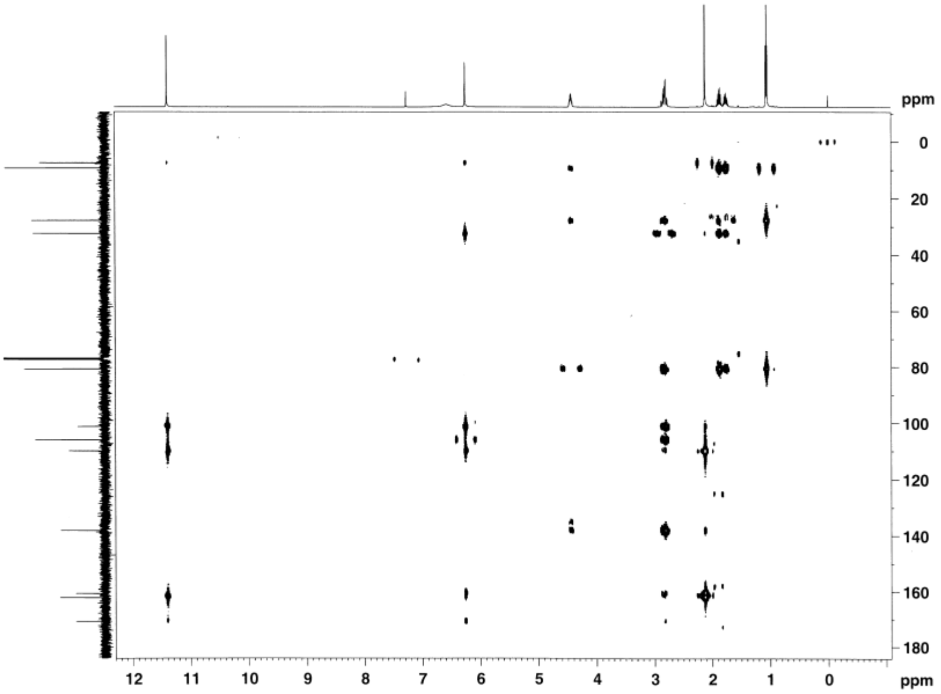

Figure S8. ^{1}H-NMR in $CDCl_3$ for compound **2**.

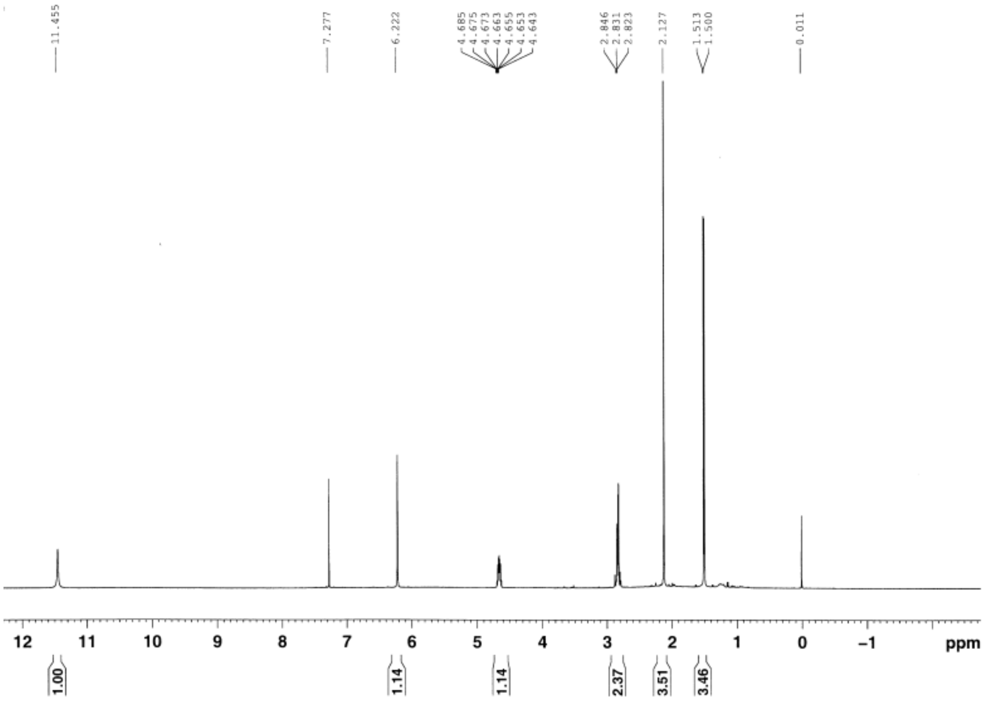

Figure S9. ^{13}C-NMR in $CDCl_3$ for compound **2**.

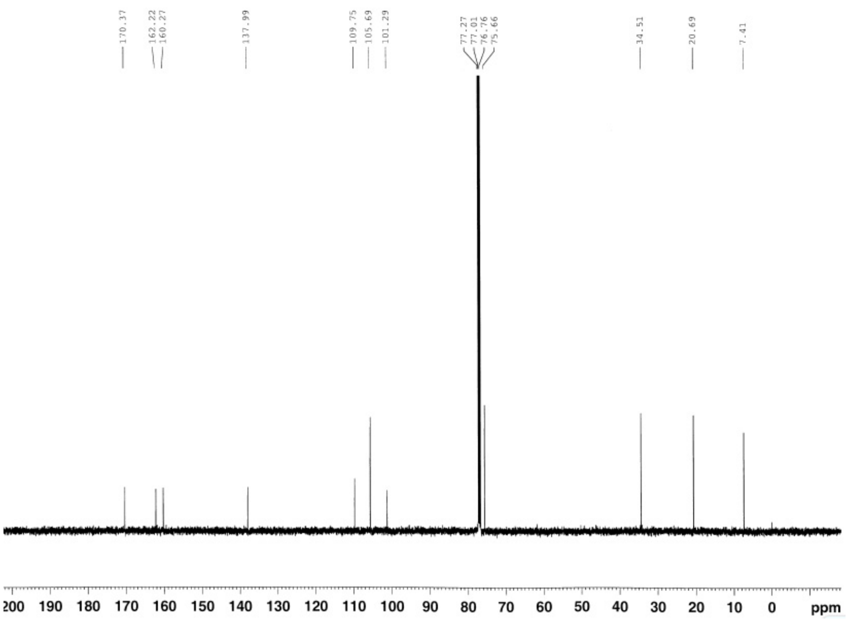

Figure S10. DEPT in $CDCl_3$ for compound **2**.

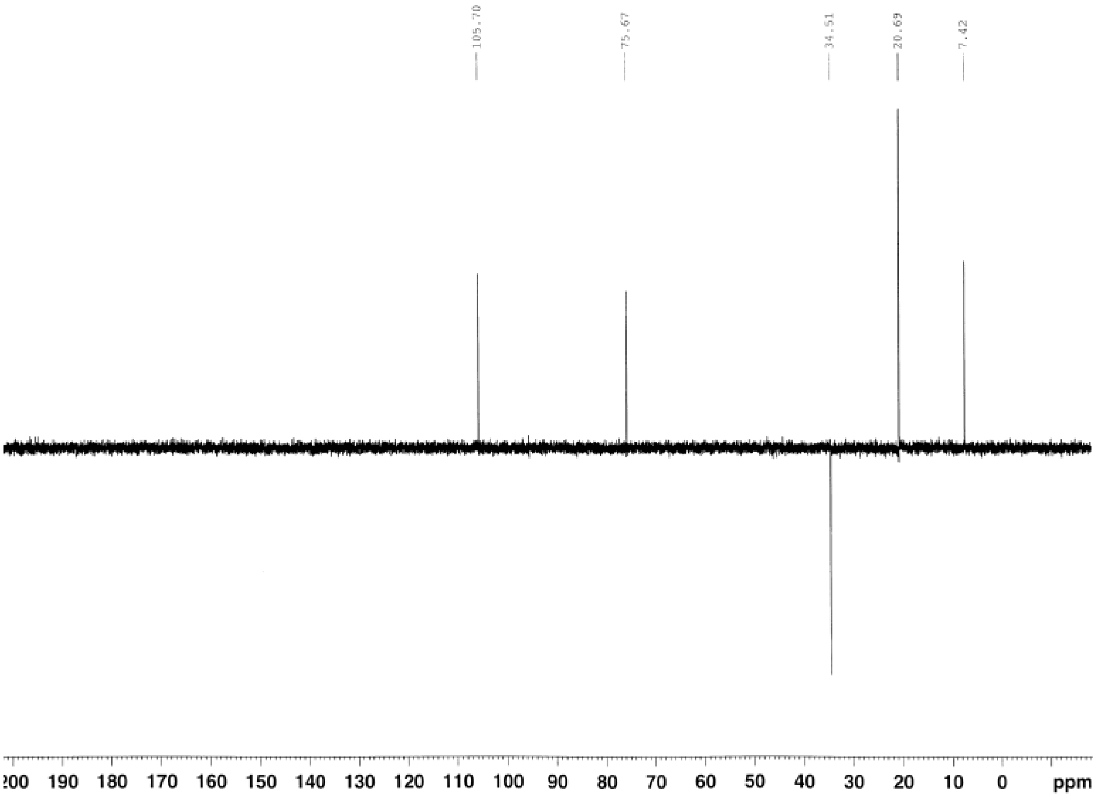

Figure S11. COSY in $CDCl_3$ for compound **2**.

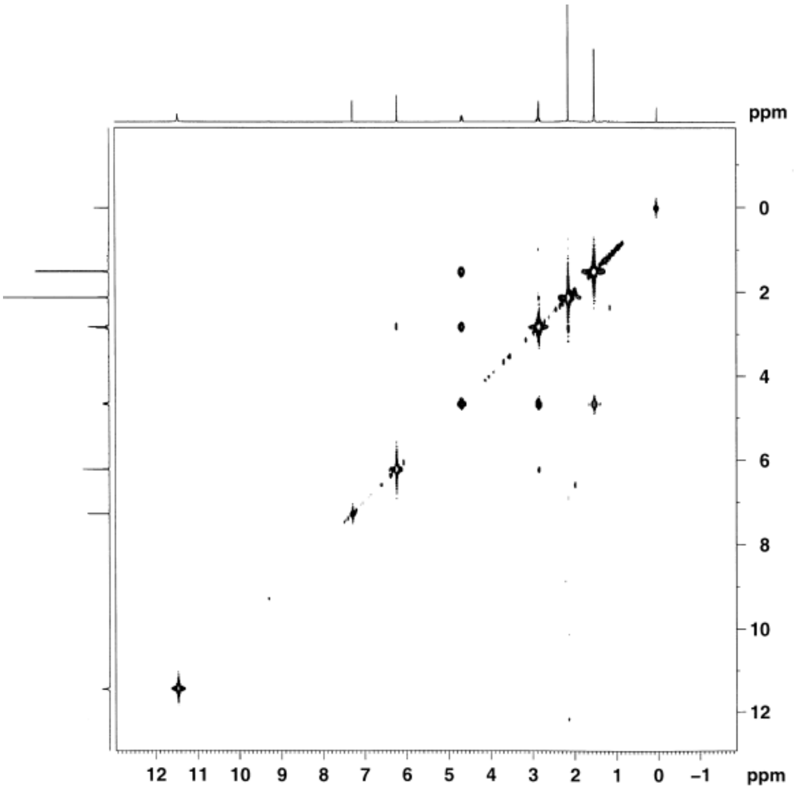

Figure S12. HMQC in $CDCl_3$ for compound **2**.

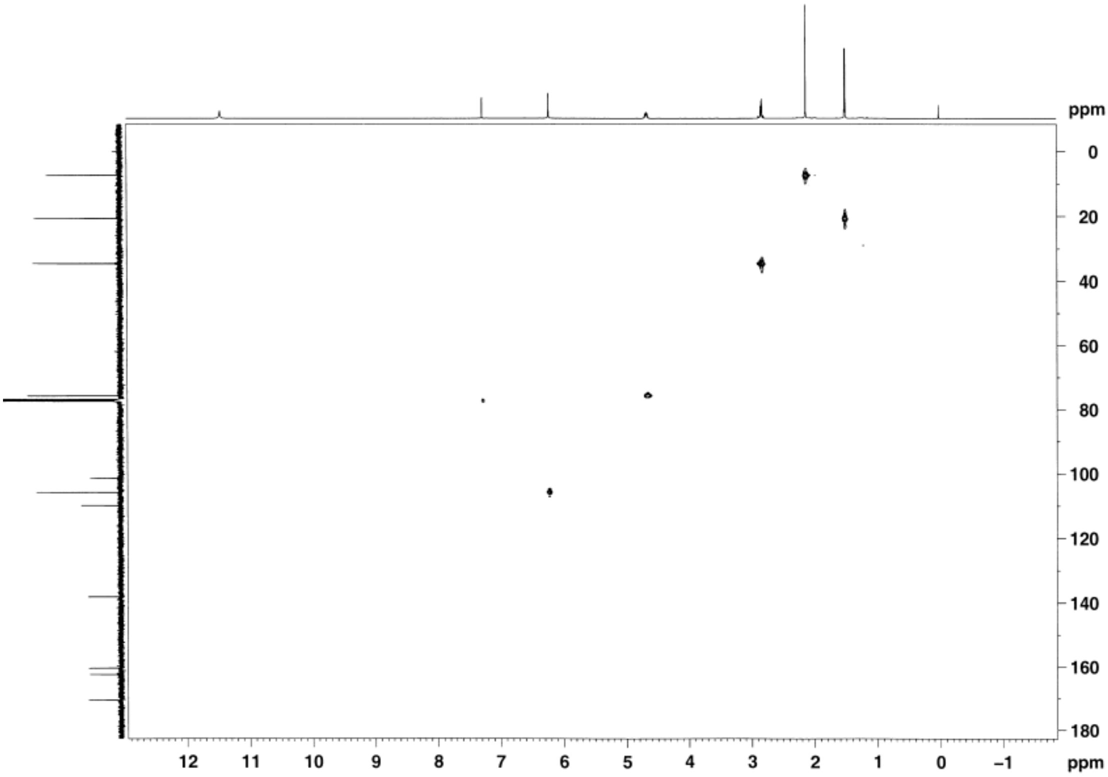

Figure S13. HMBC in $CDCl_3$ for compound **2**.

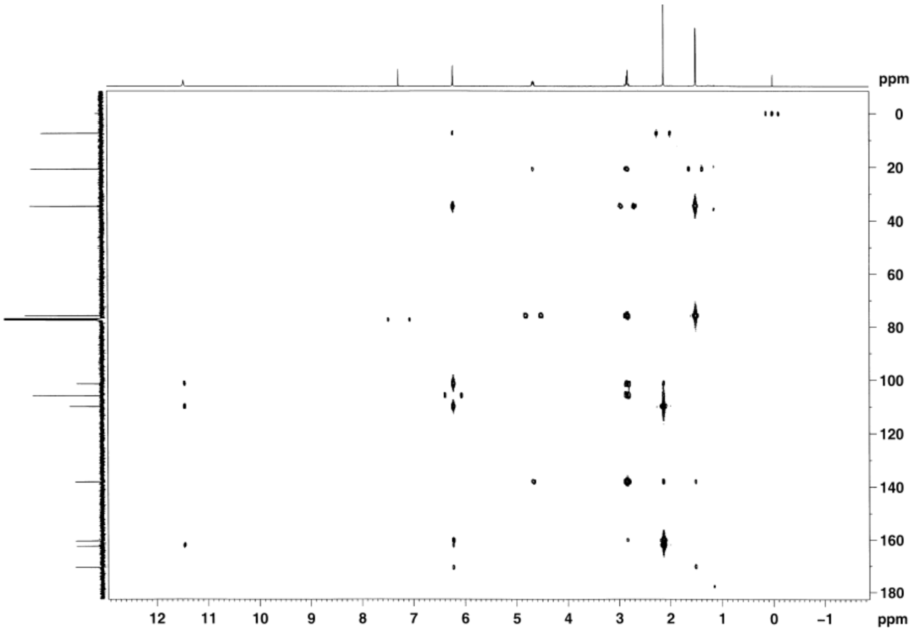

Figure S14. HR-TOF-MS for compound **1**.

Elemental Composition Report

Single Mass Analysis
Tolerance = 20.0 PPM / DBE: min = -1.5, max = 50.0
Element prediction: Off

Monoisotopic Mass, Odd and Even Electron Ions
10 formula(e) evaluated with 1 results within limits (up to 50 closest results for each mass)
Elements Used:
C: 0-20 H: 0-20 O: 0-5
09-8-1 1529 (9.905) Cm (1529:1533-1481:1500)
TOF MS CI+

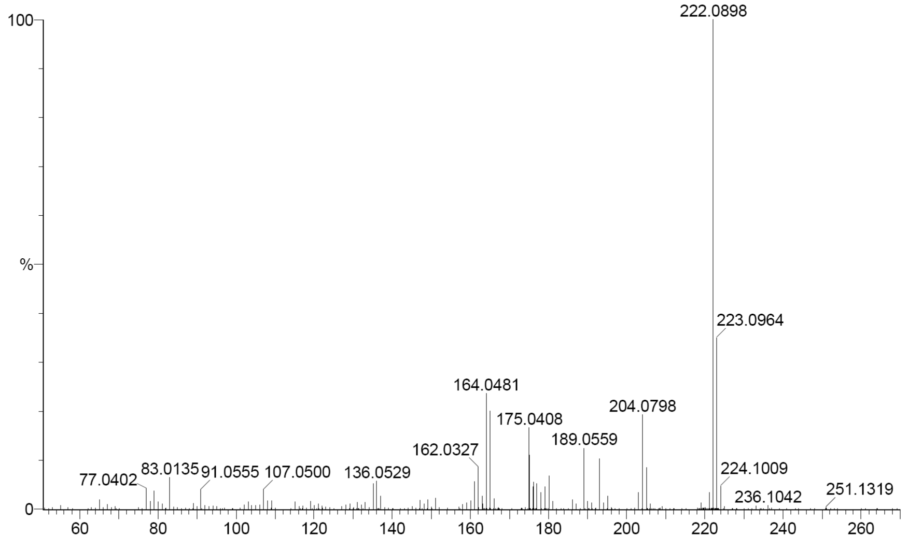

Minimum:				-1.5		
Maximum:		3.0	20.0	50.0		
Mass	Calc. Mass	mDa	PPM	DBE	i-FIT	Formula
222.0898	222.0892	0.6	2.7	6.0	676.3	C12 H14 O4

Figure S15. HR-TOF-MS for compound **2**.

Single Mass Analysis
Tolerance = 20.0 PPM / DBE: min = -1.5, max = 50.0
Element prediction: Off

Monoisotopic Mass, Odd and Even Electron Ions
12 formula(e) evaluated with 1 results within limits (up to 50 closest results for each mass)
Elements Used:
C: 0-20 H: 0-20 O: 0-5
09-8-1 1457 (9.545) Cm (1455:1462-1411:1435)
TOF MS CI+

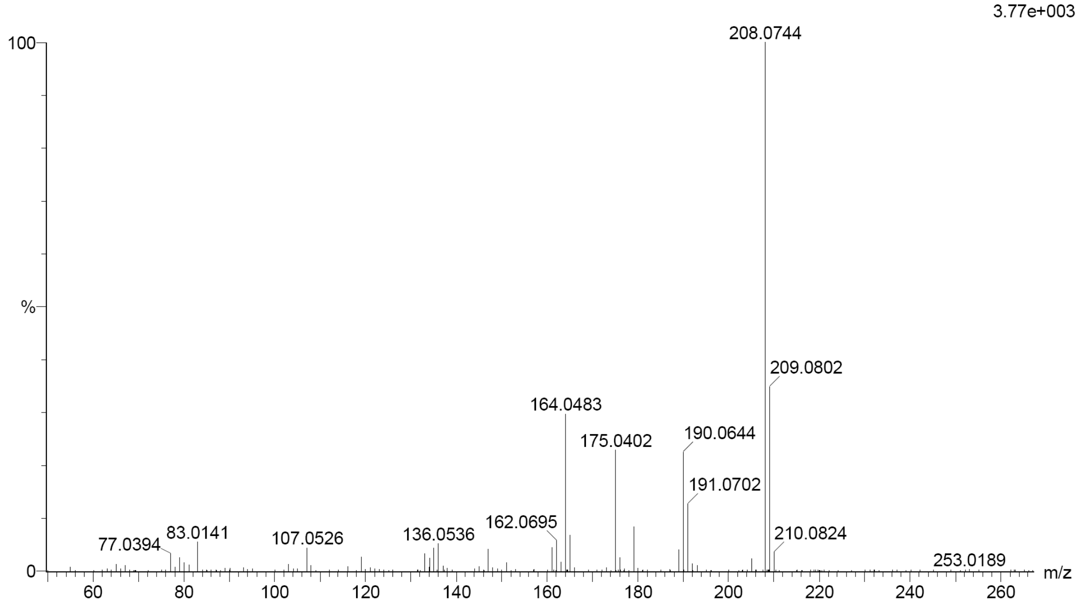

Minimum:				-1.5		
Maximum:		3.0	20.0	50.0		
Mass	Calc. Mass	mDa	PPM	DBE	i-FIT	Formula
208.0744	208.0736	0.8	3.8	6.0	274.1	C11 H12 O4

Figure S16. CCC chromatogram

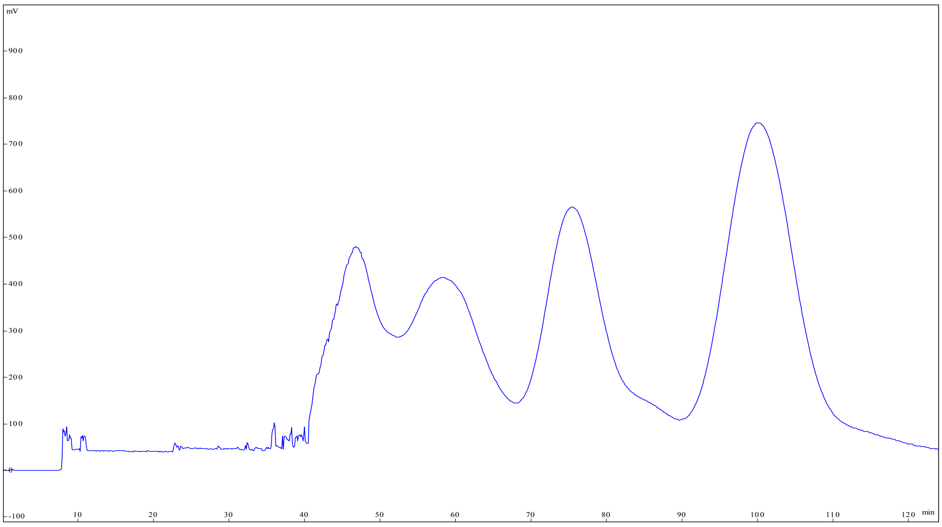

Figure S17. CD spectrum for compound **1**.

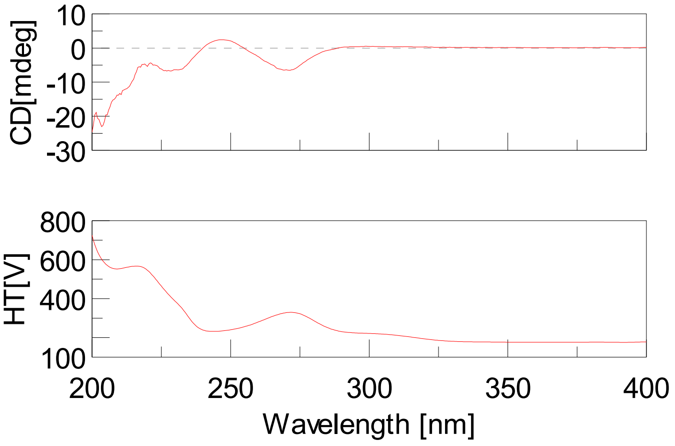

Figure S18. CD spectrum for compound **2**.

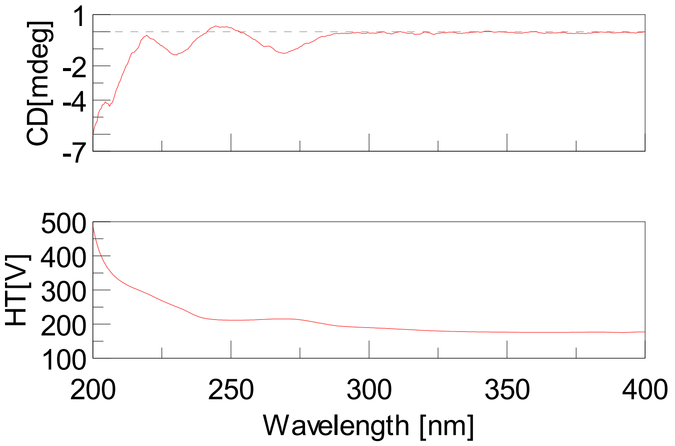

Supplement: Supplementary file 1 [file molecules-18-11299-s001.pdf]
